# Whole‐Exome Sequencing and Experimental Validation Unveil the Roles of TMEM229A Q200del Mutation in Lung Adenocarcinoma

**DOI:** 10.1111/crj.70006

**Published:** 2024-08-26

**Authors:** Yi‐Xian Liang, Yan‐Ping Xie, Huan‐Ming Yu, Wen‐Juan Zhu, Cheng‐Yi Yin, Zhao‐Hui Dong, Xi‐Lin Zhang

**Affiliations:** ^1^ Department of Cardiothoracic Surgery First Affiliated Hospital of Huzhou University Huzhou Zhejiang People's Republic of China; ^2^ Department of Respiratory Medicine First Affiliated Hospital of Huzhou University Huzhou Zhejiang People's Republic of China; ^3^ Department of Pathology The First People's Hospital of Huzhou Huzhou Zhejiang People's Republic of China; ^4^ Central Laboratory, Huzhou Key Laboratory of Translational Medicine First Affiliated Hospital of Huzhou University Huzhou Zhejiang People's Republic of China

**Keywords:** lung adenocarcinoma, micropapillary component, mutation, transmembrane protein 229A, whole‐exome sequencing

## Abstract

**Introduction:**

Lung adenocarcinoma (LUAD) is one of the major histopathological types of non‐small cell lung cancer (NSCLC), including solid, acinar, lepidic, papillary and micropapillary subtypes. Increasing evidence has shown that micropapillary LUAD is positively associated with a higher percentage of driver gene mutations, a higher incidence of metastasis and a poorer prognosis, while lepidic LUAD has a relatively better prognosis. However, the novel genetic change and its underlying mechanism in the progression of micropapillary LUAD have not been exactly determined.

**Methods:**

A total of 181 patients with LUAD who underwent surgery at the First Affiliated Hospital of Huzhou University from January 2020 to December 2022 were enrolled. Three predominant lepidic and three predominant micropapillary LUAD tissue samples were carried out using whole‐exome sequencing. Comprehensive analysis of genomic variations and the difference between lepidic and micropapillary LUAD was performed. In addition, the *TMEM229A* Q200del mutation was verified using our cohort and TCGA‐LUAD datasets. The correlations between the *TMEM229A* Q200del mutation and the clinicopathological characteristics of patients with LUAD were further analyzed. The functions and mechanisms of *TMEM229A* Q200del on NSCLC cell proliferation and migration were also determined.

**Results:**

The frequency of genomic changes in patients with micropapillary LUAD was higher than that in patients with lepidic LUAD. Mutations in *EGFR*, *ATXN2*, *C14orf180*, *MUC12*, *NOTCH1*, and *PKD1L2* were concomitantly detected in three predominant micropapillary and three predominant lepidic LUAD cases. The *TMEM229A* Q200del mutation was only mutated in lepidic LUAD. Additionally, the *TMEM229A* Q200del mutation had occurred in 16 (8.8%) patients, and not found *TMEM229A* R76H and M346T mutations in our cohort, while *TMEM229A* mutations (R76H, M346T, and Q200del) occurred only in 1.0% of the TCGA‐LUAD cohort. Further correlation analysis between the *TMEM229A* Q200del mutation and clinicopathological characteristics suggested that a lower frequency of the Q200del mutation was significantly associated with positive lymph node metastasis, advanced TNM stage, positive cancer thrombus, and pathological features. Finally, overexpression of *TMEM229A* Q200del suppressed NSCLC cell proliferation and migration in vitro. Mechanistically, overexpression of TMEM229A and TMEM229A Q200del both reduced the expression level of phosphorylated (p)‐ERK and p‐AKT (Ser473), and the reduced protein level of p‐ERK in the TMEM229A Q200del group was more pronounced compared to the TMEM229A group.

**Conclusion:**

Our results demonstrated that the *TMEM229A* Q200del mutant may play a protective role in the progression of LUAD via inactivating ERK pathway, providing a potential therapeutic target in LUAD.

## Graphical abstract

The *TMEM229A* Q200del mutation had occurred in 16 (8.8%) patients and not found *TMEM229A* R76H and M346T mutations in our cohort. Further correlation analysis between the *TMEM229A* Q200del mutation and clinicopathological characteristics suggested that a lower frequency of the Q200del mutation was significantly associated with positive lymph node metastasis, advanced TNM stage, positive cancer thrombus, and pathological features. Overexpression of *TMEM229A* Q200del suppressed NSCLC cell proliferation and migration in vitro. Mechanistically, overexpression of TMEM229A and TMEM229A Q200del both reduced the expression level of phosphorylated (p)‐ERK and p‐AKT (Ser473), and the reduced protein level of p‐ERK in the TMEM229A Q200del group was more pronounced compared to the TMEM229A group.

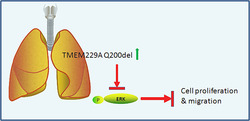

**Abbreviations**
ALK: anaplastic lymphoma kinase
ARMS: amplification refractory mutation system
ATS: American Thoracic Society
CRISPR: Clustered Regularly Interspaced Short Palindromic Repeats
DBS: double base substitution
EGFR: epidermal growth factor receptor
ERK: extracellular signal–regulated kinase
ERS: European Respiratory Society
ExAC: the Exome Aggregation Consortium
FFPE: formalin‐fixed paraffin‐embedded
gnomAD: the Genome Aggregation Database
IASLC: International Association for the Study of Lung Cancer
InDel: small insertions and deletions
LUAD: lung adenocarcinoma
NSCLC: non‐small cell lung cancer
ROS1: ROS proto‐oncogene 1 receptor tyrosine kinase
RTCA: real‐time cellular analysis
SBS: sequencing by synthesis
SBS: single base substitution
SNV: single nucleotide variant
TCGA: The Cancer Genome Atlas Program
TMEM229A: transmembrane protein 229A
TNM: tumor‐node‐metastasis
WHO: World Health Organization

## 1 Introduction

Lung adenocarcinoma (LUAD) is the most prevalent pathological subtype of lung cancer and exhibits diverse histological patterns and molecular characteristics, constituting approximately 60% of all lung cancer cases [1]. In 2015, the World Health Organization (WHO) primarily categorized LUAD into five histological classifications, namely solid, acinar, lepidic, papillary, and micropapillary pattern types [2]. Additionally, micropapillary LUAD cases are nonpredominant micropapillary adenocarcinomas. Previous study has shown that LUAD patients with micropapillary components often have lymphovascular invasion and pleural invasion, as well as lymph node or intrapulmonary metastasis features, and manifest more aggressive behavior and poorer outcomes than that other LUAD pattern types [3].

In consideration of the explanation for the mechanisms beyond tumorigenesis and malignancy discrepancy, several studies have been conducted to evaluate the molecular mechanisms and genetic changes of the LUAD subtype, particularly focusing on micropapillary LUAD [4, 5]. Recent advance have revealed that patients with micropapillary components exhibited disruption of the catenin‐cadherin complex, which contributed to its intracellular adherence [6, 7]. In addition, our previous study demonstrated that micropapillary LUAD has a significantly higher tumor mutation burden, such as *EGFR* mutations and *ROS1* fusions. The incidence of coexisting *EGFR* mutations and *ROS1* fusions is higher in this subtype compared to other subtypes of LUAD [8]. Warth et al. [9] also found that patients with micropapillary structure had a significantly higher tumor gene mutation burden and rearrangements of *ROS1* or *ALK* than other histological subtypes of LUAD. Although specific genetic alterations associated with poorer prognosis of patients with micropapillary LUAD were identified, novel gene mutations and their related key mechanisms are not fully understood.

The transmembrane protein (TMEM) family genes are located in the different biological membranes of the cell and performed a wide variety of roles in physiological and pathological phenomena [10]. TMEM229A is a member of the TMEM family that plays an important role in tooth differentiation and development [11]. In addition, the single nucleotide variant (SNP) of *TMEM229A* rs7783359 was associated with sport performance [12, 13]. Our previous study also indicated that elevated TMEM229A suppressed NSCLC progression via inactivating ERK (extracellular signal–regulated kinase) pathway [14], but the function of TMEM229A mutants in LUAD is unknown.

In the present study, we aimed to investigate the evolutionary trajectory between LUAD histological subtypes and screen subtype‐specific genetic changes. In addition, we further studied the roles and mechanisms of *TMEM229A* Q200del mutant in the progression of LUAD, providing a novel therapeutic target for the treatment of LUAD, particularly in micropapillary LUAD.

## 2 Materials and Methods

### 2.1 Patient Samples

In the present study, all patients diagnosed with LUAD at the First Affiliated Hospital of Huzhou University from January 2020 to December 2022 were enrolled. Those patients diagnosed with LUAD were selected, but patients receiving presurgical therapy or combining other malignancies were excluded. The pathological diagnosis was confirmed using hematoxylin and eosin staining by two experienced pathologists (Qilin Shi and Hui Xia from the First Affiliated Hospital of Huzhou University). In the selection criteria, a total of 181 patients were enrolled. Among them, 54 cases harbored more than 5% of the micropapillary component, and the remaining cases were other LUAD subtypes according to the International Association for the Study of Lung Cancer (IASLC)/American Thoracic Society (ATS)/European Respiratory Society (ERS) [15], including 45 solid, 36 acinar, 28 lepidic, and 18 papillary subtypes. The clinicopathological characteristics collected, including sex, age, smoking history, tumor size, tumor differentiation, tumor‐node‐metastasis (TNM) stage, cancer thrombus, and lymph node metastasis. This study was approved by the Ethics Committee of the First Affiliated Hospital of Huzhou University (approved number: 2020KYLL049). Informed consent was obtained from all patients. The clinicopathological characteristics of all patients are shown in Table 1.

**TABLE 1:** Association between *TMEM229A* Q200del mutation and clinicopathological features of patients with lung adenocarcinoma.

Variables	TMEM229A		*χ* ^2^	*p*
	WT (*n* = 165)	Q200del (*n* = 16)		
Gender			4.242	0.039*
Female	75 (45.5%)	3 (18.8%)		
Male	90 (54.5%)	13 (81.2%)		
Age			2.103	0.147
> 65	57 (34.5%)	9 (56.3%)		
≤ 65	108 (65.5%)	7 (43.7%)		
Smoking history			0.494	0.482
Ever	88 (53.3%)	10 (62.5%)		
Never	77 (46.7%)	6 (37.5%)		
Tumor size (cm)			0.155	0.693
> 3.0	44 (26.7%)	5 (31.3%)		
≤ 3.0	121 (73.3%)	11 (68.7%)		
Tumor differentiation			0.155	0.693
Well/moderate	116 (70.3%)	12 (75.0%)		
Poor	49 (29.7%)	4 (25.0%)		
Lymphatic invasion			4.258	0.043*
Present	50 (30.3%)	2 (12.5%)		
Absent	115 (69.7%)	14 (87.5%)		
TNM stage			4.459	0.035*
I + II	131 (79.4%)	9 (56.3%)		
III + IV	34 (20.6%)	7 (43.7%)		
Cancer thrombus			4.174	0.044*
Present	36 (21.8%)	1 (6.3%)		
Absent	129 (78.2%)	15 (93.7%)		
Histological type			9.152	0.002**
Micropapillary	53 (32.1%)	1 (6.3%)		
Papillary	14 (8.5%)	4 (25.0%)		
Solid	43 (26.1%)	2 (12.5%)		
Acinar	32(19.4%)	4 (25.0%)		
Lepidic	23 (13.9%)	5 (31.2%)		

* *p* < 0.05,

** *p* < 0.01.

### 2.2 DNA Extraction and Quantification

DNA extraction and purification from formalin‐fixed paraffin‐embedded (FFPE) tissues were performed using a commercial kit (cat. no. 56404, QIAamp DNA FFPE Tissue Kit, Qiagen, Germany) according to the manufacturer's protocol. Briefly, 5‐μm‐thick FFPE samples were dewaxed, and xylene was removed. After the samples were lysed, washed, and purified, the quantity and quality of DNA were eluted and measured using a NanoDrop 2000.

### 2.3 Whole‐Exome Sequencing

In all selected patients, three micropapillary LUAD (more than 50% of the micropapillary component, namely, served as predominant micropapillary LUAD) and three lepidic LUAD (more than 50% of the lepidic component, namely served as predominant lepidic LUAD) cases were selected, and whole‐exome sequencing was carried out using the Illumina HiSeq X Ten sequencing platform (Origingene of Biotechnology, Shanghai). Briefly, the sequencing libraries were further constructed by an Illumina Pair‐end (PE). Later, sequencing was performed based on sequencing by synthesis (SBS). Raw sequencing data were generated in a FASTQ file format and principally filtered on the total read volume, GC content, Q20 and Q30 percentage, and duplication rate. In addition, the sequencing data quality was assessed using the software FastQC (version 0.11.7), and the data were then compared with the human reference genome (hg19) using BWA software (https://ccb.jhu.edu/software/hisat2/ index.shtml). To ensure the reliability of the data, we clarified the criteria for the identifying and filtering on single nucleotide variants (SNVs), including (1) mutations with a number of supporting reads greater than 20 were saved; (2) mutations with non‐zero variant allele frequency (VAF) (> 0.1) were removed; (3) variants with low frequency (≤ 0.01) from the 1000 Genomes Project (https://www.internationalgenome.org/), the Genome Aggregation Database (gnomAD) (https://gnomad.broadinstitute.org), and the Exome Aggregation Consortium (ExAC) were retained; and (4) only functional variants were presented. After sample coverage filtration, SNVs and small insertions and deletions (InDels) were identified by MuTect (version 1.14) from GATK (version 4.0) [16]. In addition, oncogenic driver genes were downloaded from the Cancer Gene Census in the COSMIC database (https://cancer.sanger.ac.uk/census), and then the mutational spectrum and absolute contribution of COSMIC v3 SBS (single base substitution) mutational characteristics were obtained by MutationalPatterns [17] on unfiltered somatic mutations, while the absolute exposures of COSMIC v3 DBS (double base substitution) InDel signatures were carried out by Sigminer [18].

### 2.4 Polymerase Chain Reaction (PCR)

PCR assays were performed using PrimeSTAR Max DNA Polymerase (cat. no. R045A, Takara Biotechnology Co. Ltd.) according to the manufacturer's protocol. The thermocycling conditions used for the PCR were as follows: initial denaturation at 95°C for 5 min, followed by 35 cycles at 94°C for 30 s, 58°C for 30 s and 72°C for 30 s, and extension at 72°C for 5 min. To construct the human TMEM229A mutants (accession no. NM_001136002.2), the specific primer sequences used in the experiment were as follows: *TMEM229A* Q200del forward, 5′‐CCTTCGTCTTCAATTTCCTCC‐3′ and reverse, 5′‐GCTGTA GTGGAGGTGGAAGTAGAG‐3′. *TMEM229A* R76H forward, 5′‐GCTGTCCACTG CTGAAGCGC‐3′ and reverse, 5′‐CGCTGCTGCAGGTACACCTT‐3′. *TMEM229A* M346T forward, 5′‐GGTGCCCATCTACGTGATCT‐3′ and reverse, 5′‐TTAGTTAGCTGGTACGTACTGC‐3′.

### 2.5 DNA Electrophoresis and Identification

The size of the PCR product (Q200del, 554 bp; R76H, 222 bp; and M346T, 217 bp, respectively) was identified by DNA electrophoresis. Later, the PCR product was purified using a TIANgel Midi Purification Kit (cat. no. #DP209‐03, Tiangen Biochemical Technology Co. Ltd.). All purification products were sequenced and identified by Shanghai Sangon Biotech Co. Ltd. (Shanghai).

### 2.6 TCGA Databases

The function and clinical significance of *TMEM229A* mutations in LUAD were explored using TCGA databases (http://www.cbioportal.org). Fifteen studies and 6936 patients with LUAD were enrolled in the study.

### 2.7 Cell Culture

Two NSCLC cell lines (A549 and H23) were purchased from the Shanghai Cell Bank of the Chinese Academy of Sciences (Shanghai, China). Cells were cultured in Dulbecco's Modified Eagle Medium (DMEM, cat. no. L110KJ, BasalMedia, Shanghai) supplemented with 10% FBS (Gibco; HyClone; Cytiva), 100 μg/mL streptomycin and 100 IU/mL penicillin (Sigma‐Aldrich; Merck KGaA) in a humidified incubator under 5% CO_2_ at 37°C.

### 2.8 Plasmid Construction and Cell Transfection

To construct the full‐length human TMEM229A proteins (accession no. NM_001136002.2), the specific primer was designed, and the corresponding TMEM229A DNA was cloned and inserted into a pcDNA3.1‐myc/his vector (abbreviation: vector; cat. no. V800‐20; Invitrogen; Thermo Fisher Scientific Inc.). The recombinant plasmids were referred to as pcDNA3.1‐TMEM229A‐*myc*/his (abbreviation: OE‐TMEM229A). For pcDNA3.1‐TMEM229A‐Q200del‐*myc*/his mutant plasmid, we directly synthesized from Shanghai Sangon Biotech Co. Ltd. according to OE‐TMEM229A plasmid. Once cells reached approximately 80% confluency, transfections were carried out using Lipofectamine 2000 reagent (Invitrogen; Thermo Fisher Scientific Inc.), according to the manufacturer's instructions. The primer sequence used in the experiment was as follows: TMEM229A forward, 5′‐CCCAAGCTTGCCACCATGGCGGGGAGCG‐3′ and reverse, 5′‐CGCGGATCCGGTTAGCTGGTACGTACTGCAC‐3′.

### 2.9 Real‐Time Cellular Analysis (RTCA)

The RTCA xCELLLigence system (ACEA Biosciences Inc.; Agilent Technologies Inc.) was widely used to monitor cell morphology, proliferation, and migration in a noninvasive procedure [19]. A cell index was served as indicate the cell number and cell adhesion. Upon cells adhered to the surface of the E‐16 plate or CIM plate, an electronic record was changed and converted into the cell index by the xCELLLigence system. After cells transfected with different plasmids, cell proliferation and migration were carried out using RTCA assay as previously described [20]. Briefly, for cell proliferation assays, 50‐μL culture medium was added to measure the background, and then 100‐μL culture medium containing 6 × 10^3^ A549 cells and 1 × 10^4^ H23 cells were seeded into the E‐16 plate. For cell migration assays, the CIM plate was consisted of the lower chamber (165‐μL culture medium was added) and the upper chamber (30‐μL serum‐free culture medium was added) and left to stand for 1 h in a humidified incubator and then measured the background. Subsequently, the cells (6–10× 10^4^) were mixed with serum‐free culture medium and seeded into the CIM plate. The data were recorded and analyzed using xCELLLigence software 2.0 (ACEA Biosciences Inc.; Agilent Technologies Inc.) [19].

### 2.10 RNA Extraction and Reverse Transcription‐Quantitative PCR

Total RNA was isolated using FastPure Cell/Tissue Total RNA Isolation Kit (cat. no. RC112‐01, Nanjing Vazyme Biotech Co. Ltd.), according to the manufacturer's protocol. Briefly, cells were lysed in Buffer RL, and the genomic DNA was removed using FastPure gDNA‐Filter columns III. Subsequently, RNA was dissolved in ethanol and washed with Buffer RW1 and Buffer RW2. Finally, the purified RNA was dissolved in RNase‐free ddH_2_O. The RNA was reverse transcribed into cDNA using the PrimeScript RT reagent kit (Takara Biotechnology Co. Ltd.), as previously described [21]. Reverse transcription‐quantitative PCR was performed following the instructions of the UltraSYBR Green PCR Master mix (cat. no. CW0957H; CWBio) on an ABI 7500 system (Applied Biosystems; Thermo Fisher Scientific Inc.). 18S ribosomal RNA (18sRNA) was used an endogenous housekeeping gene for normalization, and the relative expression level was calculated using the 2^−ΔΔCq^ method [22]. The primer sequences used in the experiment were as follows: TMEM229A forward, 5′‐CGACCTACCCCGCTTTCTTTT‐3′ and reverse, 5′‐GCTCCCACCGTAGATGAAGAT‐3′; and 18sRNA forward, 5′‐GTAACCCGTTGAACCCCATT‐3′ and reverse, 5′‐CCATC CAATCGGTAGTAGCG‐3′.

### 2.11 Western Blotting

Total protein was extracted using radioimmunoprecipitation (RIPA) buffer containing protein and phosphatase inhibitors (Beyotime Institute of Biotechnology, Shanghai) according to our previous reports [14, 20]. An equal amount of protein was separated using 4%–20% SDS‐PAGE, and then the proteins were transferred onto 0.45‐μm PVDF membranes (EMD Millipore), followed by blocking with 5% bovine serum albumin (BSA) at room temperature for 1 h. The membranes were incubated with primary antibodies overnight at 4°C. After washing with PBS containing 0.1% Tween‐20, the membranes were incubated with the corresponding HRP‐conjugated secondary antibodies ([goat‐anti rabbit IgG (H + L) antibody; 1:2000; cat. no. A0208; Beyotime Institute of Biotechnology] or [goat‐anti mouse IgG (H + L) antibody; 1:2000; cat. no. A0216; Beyotime Institute of Biotechnology]) at room temperature for 1 h. The immunoblots were visualized using an ECL reagent (cat. no. P0018S; Beyotime Institute of Biotechnology) and detected by a Tanon 5200 system (Tanon Science & Technology Co. Ltd.). β‐actin was used an endogenous housekeeping gene for normalization, and the relative expression level of proteins was calculated using ImageJ v1.6.0 software (National Institutes of Health). The primary antibodies used in this experiment were as follows: anti‐TMEM229A (1:500; cat. no. ab107780; Abcam); anti‐ERK1/2 (1:2000; cat. no. 4695; Cell Signaling Technology Inc.); anti‐phosphorylated (p)‐ERK1/2 (1:2000; cat. no. 4370; Cell Signaling Technology Inc.); anti‐AKT (1:2000; cat. no. 4691; Cell Signaling Technology Inc.); anti‐p‐AKT (1:2000; Ser473; cat. no. 4060; Cell Signaling Technology Inc.); and anti‐β‐actin (1:5000; cat. no. M1210‐2; Hangzhou HuaAn Biotechnology Co. Ltd.).

### 2.12 Hematoxylin–Eosin (H&E) Staining

The LUAD tissues were preserved and immersed using 4% paraformaldehyde solution and then embedded in paraffin. The 5‐μm sections were stained using H&E staining solution (cat. no. C0105S; Beyotime Institute of Biotechnology). The images were visualized and analyzed under a light microscope at 100× magnification.

### 2.13 Statistical Analyses

The data were collected and analyzed using SPSS Statistics software (version 21.0; IBM Corp.). Data were presented as the mean ± standard error of mean of three independent experiments and were analyzed using an unpaired Student's *t* test or one‐way ANOVA followed by Tukey's post hoc test. Categorical variables were presented as frequencies and percentages, and variables among different groups were compared using chi‐square tests or Fisher's exact tests. *p* < 0.05 was considered statistically significant.

## 3 Results

### 3.1 Clinicopathological Characteristics of Patients

Among the 181 patients with LUAD, 54 (29.8%) had a micropapillary pattern, 45 (24.9%) had a solid pattern, 36 (19.9%) had acinar pattern, 28 (15.5%) had lepidic pattern, and 18 (9.9%) had papillary carcinoma. The clinicopathological characteristics of all patients are presented in Table 1.

### 3.2 Mutational Landscape of Micropapillary and Lepidic LUAD

**FIGURE 1:**
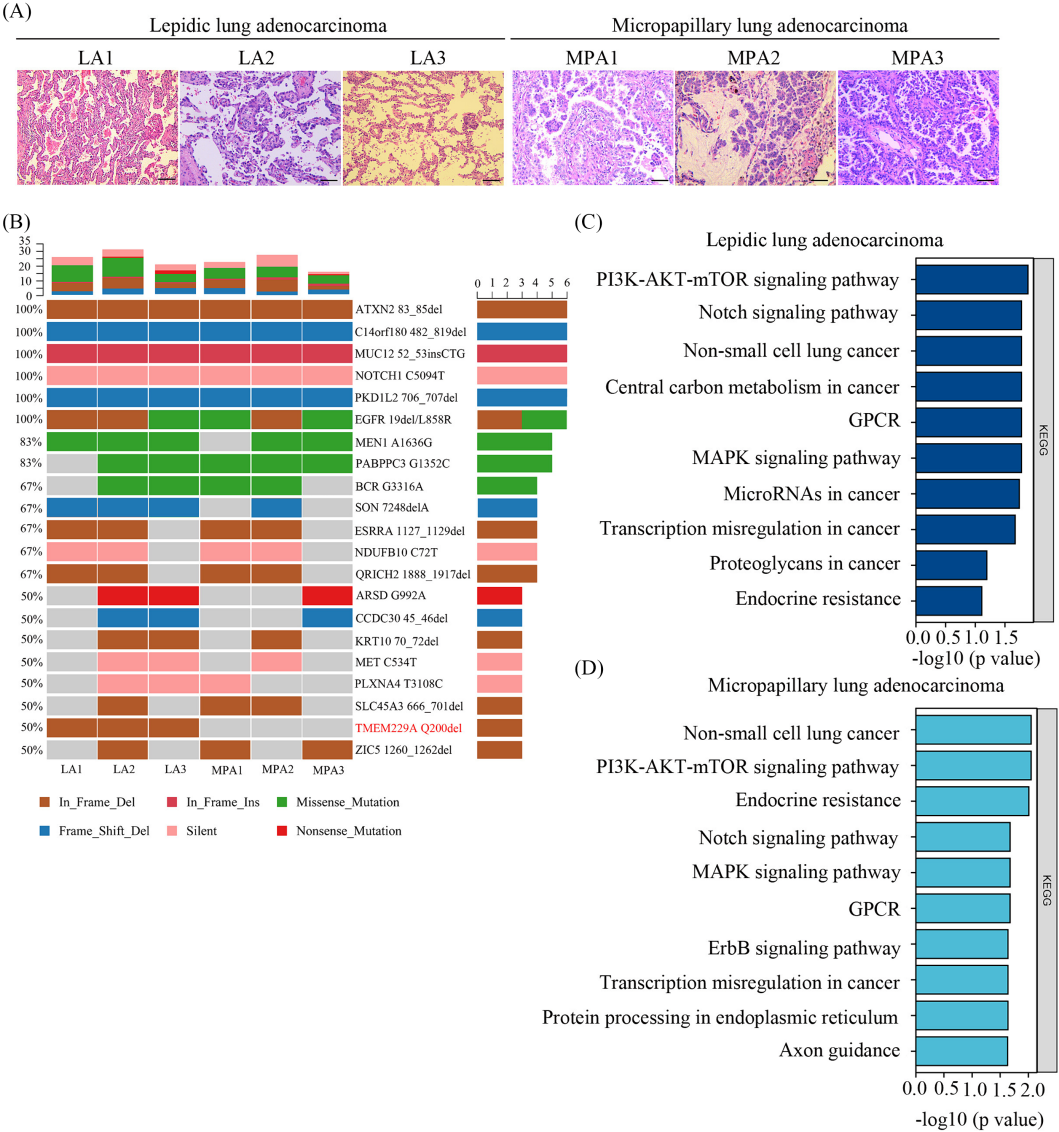
H&E‐stained slides and mutational landscape of micropapillary and lepidic lung adenocarcinoma. (A) The slides from three lepidic and three micropapillary lung adenocarcinoma patients were stained using hematoxylin–eosin staining (100× magnification). (B) Cancer‐associated genes with the top mutation in three lepidic and three micropapillary lung adenocarcinoma samples were analyzed using whole‐exome sequencing. (C) Pathways enriched in lepidic lung adenocarcinoma were analyzed by KEGG. (D) Pathways enriched in micropapillary lung adenocarcinoma were performed by KEGG.

According to a previous report, patients with micropapillary LUAD showed the worst prognosis, while lepidic LUAD had a protective role in prognosis [3]. The histological images of micropapillary and lepidic lesions are shown in Figure 1A. To explore the differences in novel gene mutations, three predominant micropapillary and three predominant lepidic LUAD patients were selected for whole‐exome sequencing. The results demonstrated that the lepidic LUAD group had 1402 gene snp and 274 gene indel mutations, but the micropapillary LUAD group had 2428 gene snp and 447 gene indel mutations, indicating that there are more gene mutations in micropapillary LUAD. In addition, *EGFR* mutations were identified as the most frequent driver gene, which was consistent with previous studies [3, 23]. Moreover, several other genes including *ATXN2*, *C14orf180*, *MUC12*, *NOTCH1*, and *PKD1L2* were concomitantly mutated in the two subtypes (Figure 1B), suggesting that the PI3K‐AKT‐mTOR, Notch, MAPK, and GPCR signaling pathways were affected (Figure 1C,D). The PI3K‐AKT‐mTOR and Notch pathways play a role in cell growth, cell apoptosis, and metastasis, which may be associated with tumor progression and therapeutic resistance. Additionally, genome‐wide association studies had shown that loss‐of‐function mutations in *ATXN2* gene may be associated with susceptibility to type I diabetes, obesity and hypertension, while *NOTCH1* gene mutations were associated with aortic value disease, Adams‐Oliver syndrome, T‐cell acute lymphoblastic leukemia, chronic lymphocytic leukemia, and head and neck squamous cell carcinoma. These data imply that lepidic and micropapillary component from LUAD were shared the same several pathway‐specific mutations. We next explored SNV and Indel mutation data and identified the gene with an alternation frequency difference between the lepidic and micropapillary LUAD. The results indicated that the *TMEM229A* Q200del mutation was only present in lepidic LUAD cases, but not in micropapillary LUAD cases (Figure 1B). However, there was no other gene showed an alternation frequency difference between the two groups.

### 3.3 Mutational Landscape of TMEM229A in LUAD

**FIGURE 2:**
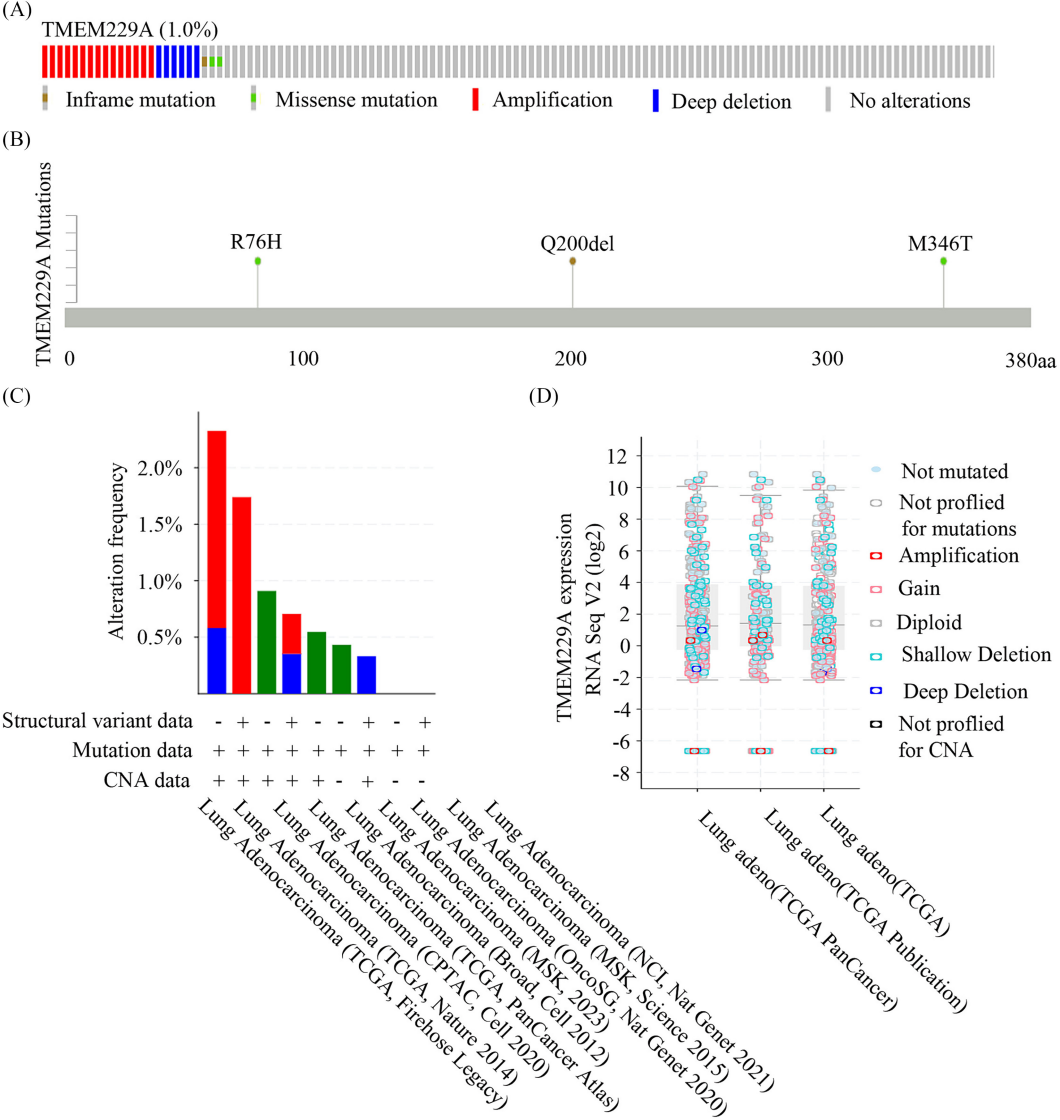
Mutational landscape of *TMEM229A* in lung adenocarcinoma. (A) The frequency of *TMEM229A* mutations in TCGA‐LUAD samples. (B) *TMEM229A* mutations (R76H, Q200del, and M346T) in TCGA‐LUAD samples. (C) The frequency of *TMEM229A* mutations in samples with Structural variant data, Mutation data, and CNA data. (D) The relationship between TMEM229A expression and TMEM229A mutations in TCGA‐LUAD samples.

To explore the clinical significance of *TMEM229A* mutations in LUAD, the distribution and roles of TMEM229A mutants in TCGA databases patients with LUAD were first analyzed, and the results indicated that 15 associated studies were included, and 6936 LUAD patients were enrolled in this study. In all selected patients, the frequency of *TMEM229A* mutations was approximately 1% (Figure 2A), namely, R76H, Q200del, and M346T (Figure 2B). Additionally, Q200del was an inframe mutation, but R76H and M346T were missense mutations. Moreover, the alteration frequency of *TMEM229A* was analyzed in nine selected studies and showed that the frequency of TMEM229A mutations was low and mainly contained structural variants, mutations, and CNA data (Figure 2C). Finally, we deeply analyzed the RNA‐seq data and found that TMEM229A expression was not changed in different *TMEM229A* variants (Figure 2D). To verify the *TMEM229A* Q200del, R76H, and M346T mutations in LUAD, specific primers were designed and synthesized, and PCR was performed. The length of the PCR product of TMEM229A Q200del was 536 bp (Figure S1A,B). In addition, the PCR product was sequenced, and the results demonstrated that 16 of 181 LUAD patients had the *TMEM229A* Q200del mutation and not found R76H and M346T mutations (Table 1). Further correlation analysis between the *TMEM229A* Q200del mutation and clinicopathological characteristics suggested that a lower frequency of the Q200del mutation was associated with positive lymph node metastasis (*p* = 0.043), advanced TNM stage (*p* = 0.035), positive cancer thrombus (*p* = 0.044), and pathological features (*p* = 0.008) (Table 1). These data suggest that the *TMEM229A* Q200del mutation may play a protective role in the tumorigenesis of LUAD.

### 3.4 TMEM229A Q200del Mutation Inhibits NSCLC Cell Proliferation and Migration via Inactivating ERK Pathway In Vitro

**FIGURE 3:**
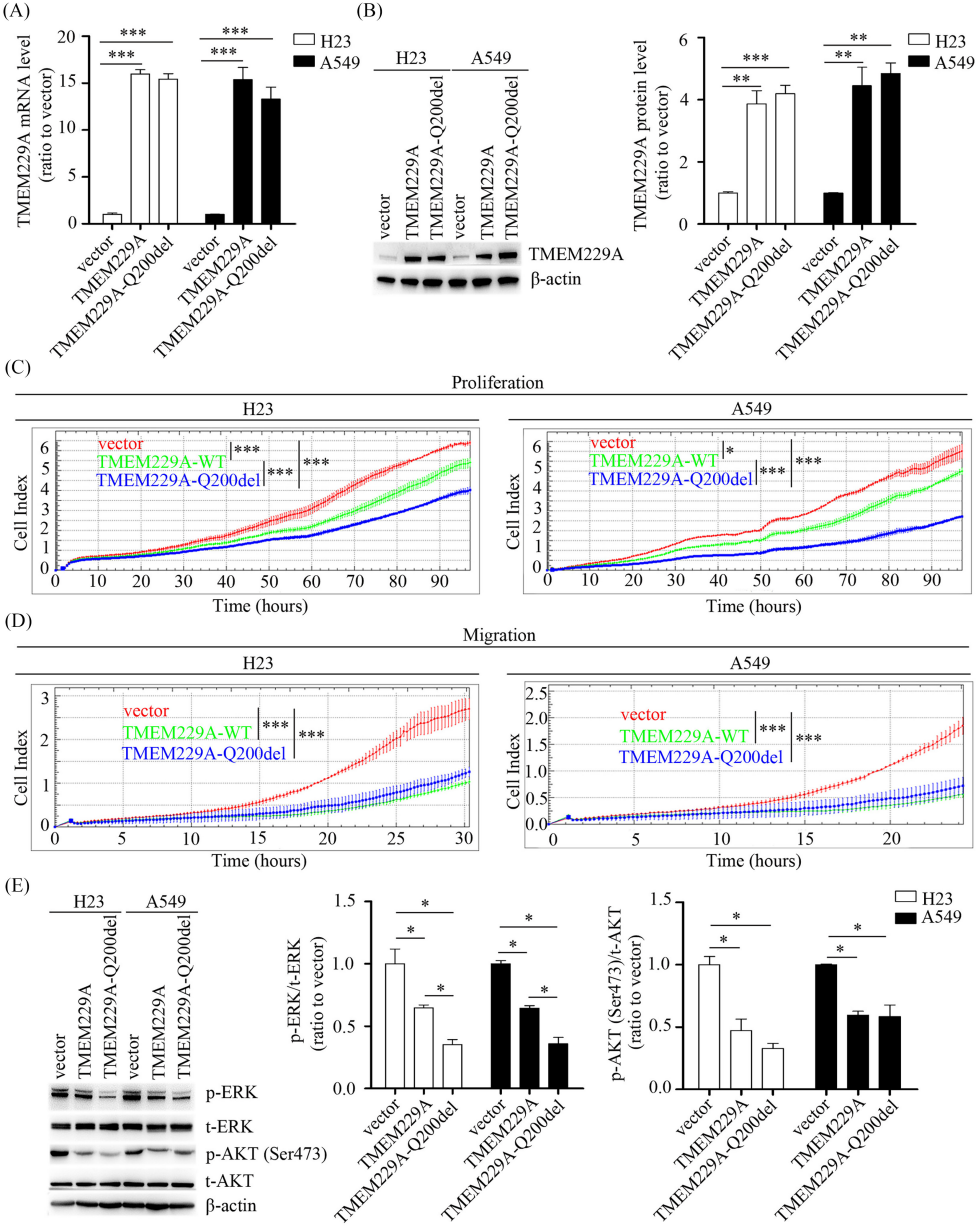
Overexpression of *TMEM229A* Q200del suppressed NSCLC cell proliferation and migration via inactivating ERK pathway. (A) H23 and A549 cells were transfected with vector, TMEM229A or TMEM229A‐Q200del for 24 h. The mRNA level of TMEM229A was measured in H23 and A549 using reverse transcription‐quantitative PCR. (B) H23 and A549 cells were transfected with vector, TMEM229A or TMEM229A‐Q200del for 48 h. The protein level of TMEM229A was detected by western blotting. β‐actin was used as a loading control. (C) After cells transfected with different plasmids, cell proliferation was assessed using RTCA assay. (D) After cells transfected with different plasmids, cell migration was performed using RTCA assay. (E) H23 and A549 cells were transfected with different plasmids. p‐ERK, t‐ERK p‐AKT Ser473, and t‐AKT were detected by western blotting. β‐actin was used as a loading control. Data represent the mean ± SEM from three independent experiments. *, *p* < 0.05; **, *p* < 0.01; ***, *p* < 0.001 were determined by one‐way ANOVA with Tukey's post hoc analysis. OE, overexpression; Q200del, TMEM229A Q200del; p, phosphorylated; t, total.

Our previous study demonstrated that TMEM229A was expressed at low levels in NSCLC and suppressed NSCLC progression by inactivating the ERK pathway, suggesting that TMEM229A is a suppressor gene in the development of NSCLC [14]. To further investigate the role of the *TMEM229A* Q200del mutation in LUAD, cell proliferation and migration assays were performed, and the results indicated that overexpression of *TMEM229A* Q200del mutant significantly upregulated TMEM229A expression and inhibited H23 and A549 (*TMEM229A* wild‐type) cell proliferation and migration in vitro (Figure 3A–D). In addition, the proliferative inhibition of *TMEM229A* Q200del mutant was more obvious than that of wild‐type TMEM229A (Figure 3C). Consistent with our previous report [14], overexpression of TMEM229A and its Q200del mutant resulted in a decrease in the expression levels of p‐ERK and p‐AKT (Ser473) (Figure 3E). Additionally, the TMEM229A Q200del group exhibited a significantly lower protein level of p‐ERK compared to the TMEM229A group (Figure 3E), indicating that the *TMEM229A* Q200del mutant inhibits LUAD progression via inactivating ERK pathway.

## 4 Discussion and Conclusions

LUAD is a morphologically heterogeneous cancer of the lung, which possesses a unique histological, radiological, epidemiological, and clinical features [24]. In 2015, the WHO mainly classified LUAD into five histological categories, including solid, acinar, lepidic, papillary and micropapillary pattern types [2]. Recent studies have demonstrated that patients with the micropapillary or solid subtype of LUAD are associated with worse outcomes due to genomic diversity [25], a higher incidence of metastasis [7], the spread of tumors through air spaces [26], and other factors [5, 27]. In addition, our previous study discovered that patients with micropapillary LUAD had a higher prevalence of EGFR mutations, ROS1 rearrangement and combined mutations of EGFR, ROS1 and EML4‐ALK using the amplification refractory mutation system (ARMS) [8], which was consistent with other previous report [28]. Based on these observations, this study intends to screen new micropapillary LUAD‐associated genes using whole‐exome sequencing and further investigate their role in the tumorigenesis of LUAD.

In the present study, the most commonly mutated driver genes were *EGFR*, *ATXN2*, *C14orf180*, *MUC12*, *NOTCH1*, and *PKD1L2* in patients with a micropapillary component and the lepidic subtype of LUAD. Previous report indicated that *EGFR* mutations are strongly associated with patients with the micropapillary subtype of LUAD [29]. In addition, most of the reported driver genes included *KARS*, *PIK3CA*, *TP53*, and *ALK* rearrangements in micropapillary LUAD [30]. Our results also found that one lepidic (1/3) and one micropapillary LUAD sample (1/3) had *TP53* mutations, but *KRAS* and *PIK3CA* mutations and ALK rearrangements were not found, which may be attributed to the small sample size. As reported previously [4], several novel gene mutations were identified in micropapillary LUAD, such as *ZNF469*, *TTN*, *TENM4*, *APOBEC*, *KEAP1*, *NOTCH4*, *PTP4A3*, *NAPRT*, and *RECQL4*. Further studies discovered that these novel genes were regarded as oncogenes or tumor suppressor genes and played a pivotal role in the progression and prognosis of LUAD. Interestingly, we observed that the *TMEM229A* Q200del mutation existed only in lepidic LUAD, but not in micropapillary LUAD, which was firstly identified. According to the UniProt databases (https://www.uniprot.org/), TMEM229A is localized to the plasma membrane and is a seven‐TMEM with poorly defined biology. A previous report showed that downregulated expression of TMEM229A contributed to the progression from deciduous to permanent teeth [11]. In addition, our previous study confirmed that TMEM229A was lowly expressed in NSCLC and partly suppressed NSCLC progression through inactivating the ERK pathway, suggesting TMEM229A was a suppressor gene in the development and progression of NSCLC [14]. A recent whole‐genome sequencing study proved that the *TMEM229A* rs7783359 polymorphism was linked explicitly with reaction time in wrestlers.^12 13^ This study showed that the *TMEM229A* Q200del mutation was associated with lymph node metastasis, TNM stage, cancer thrombus and pathological pattern. Overexpression of *TMEM229A* Q200del mutant significantly inhibited NSCLC cell proliferation and migration. Moreover, the inhibition of *TMEM229A* Q200del mutant was more obvious than that of wild‐type TMEM229A. Mechanistically, overexpression of TMEM229A and TMEM229A Q200del mutant both reduced the expression levels of p‐ERK and p‐AKT (Ser473), and the reduced expression level of p‐ERK/t‐ERK in TMEM229A Q200del group was more obvious than that in TMEM229A group, suggesting the *TMEM229A* Q200del mutant inhibits LUAD progression via inactivating ERK pathway.

Overall, our results demonstrate that the mutation burden in micropapillary LUAD was greater than that in lepidic LUAD. In addition, the *TMEM229A* Q200del mutation appeared in lepidic LUAD but not in micropapillary LUAD. Moreover, overexpression of the *TMEM229A* Q200del mutant significantly suppressed NSCLC cell proliferation and migration via inactivating ERK pathway, providing a novel therapeutic target and a promising translational marker for the treatment of LUAD, particularly in micropapillary LUAD. However, the present study had several limitations. First, the cohort only selected three micropapillary components and three lepidic subtypes of LUAD for whole‐exome sequencing. Future studies with a larger number of samples will be conducted. Additionally, the investigation of TMEM229A Q200del's function in NSCLC cell lines by plasmid overexpression does not truly represent the function of the endogenous mutation. The function of endogenous TMEM229A Q200del mutant should be further analyzed via Clustered Regularly Interspaced Short Palindromic Repeats (CRISPR). Finally, the mechanism of *TMEM229A* Q200del mutant in LUAD progression was not fully determined. Thus, we will further investigate the underlying mechanism of *TMEM229A* Q200del and TMEM229A in the development and progression of LUAD.

## Author Contributions

Xilin Zhang and Zhaohui Dong designed and conceived the study. Yixian Liang, Yanping Xie, Huanming Yu, Wenjuan Zhu, and Chengyi Yin performed these experiments. Yixian Liang, Zhaohui Dong, and Xilin Zhang analyzed the data. Xilin Zhang and Yixian Liang wrote the manuscript. All authors have read the final manuscript.

## Ethics Statement

This work was approved by the Ethics Committee of the First People's Hospital of Huzhou (approved number: 2020KYLL049), and informed consent was obtained from all patients.

## Consent

Informed consent was obtained from all patients.

## Conflicts of Interest

The authors declare no conflicts of interest.

## Supporting information


**Figure S1** *TMEM229A* Q200del genotype was identified. (A) *TMEM229A* Q200del mutation of selected 10 cases was identified using DNA electrophoresis; (B) *TMEM229A* genotype was further performed using DNA sequencing.

## Data Availability

The corresponding datasets have been submitted to Sequence Read Archive (SRA). database (BioProject ID: PRJNA1044823). The persistent URL directly linked with https://dataview.ncbi.nlm.nih.gov/object/PRJNA1044823?reviewer=ofkt4k3518m7mevjjb5mti2mjn. All datasets used and/or analyzed in this article are available from the corresponding authors upon reasonable request.
