# Supplementary figures and images for: Whole‐Exome Sequencing and Experimental Validation Unveil the Roles of TMEM229A Q200del Mutation in Lung Adenocarcinoma
Source: Clin Respir J. 2024 Aug 26;18(8):e70006. doi: 10.1111/crj.70006 (PMC11347615; doi:10.1111/crj.70006)

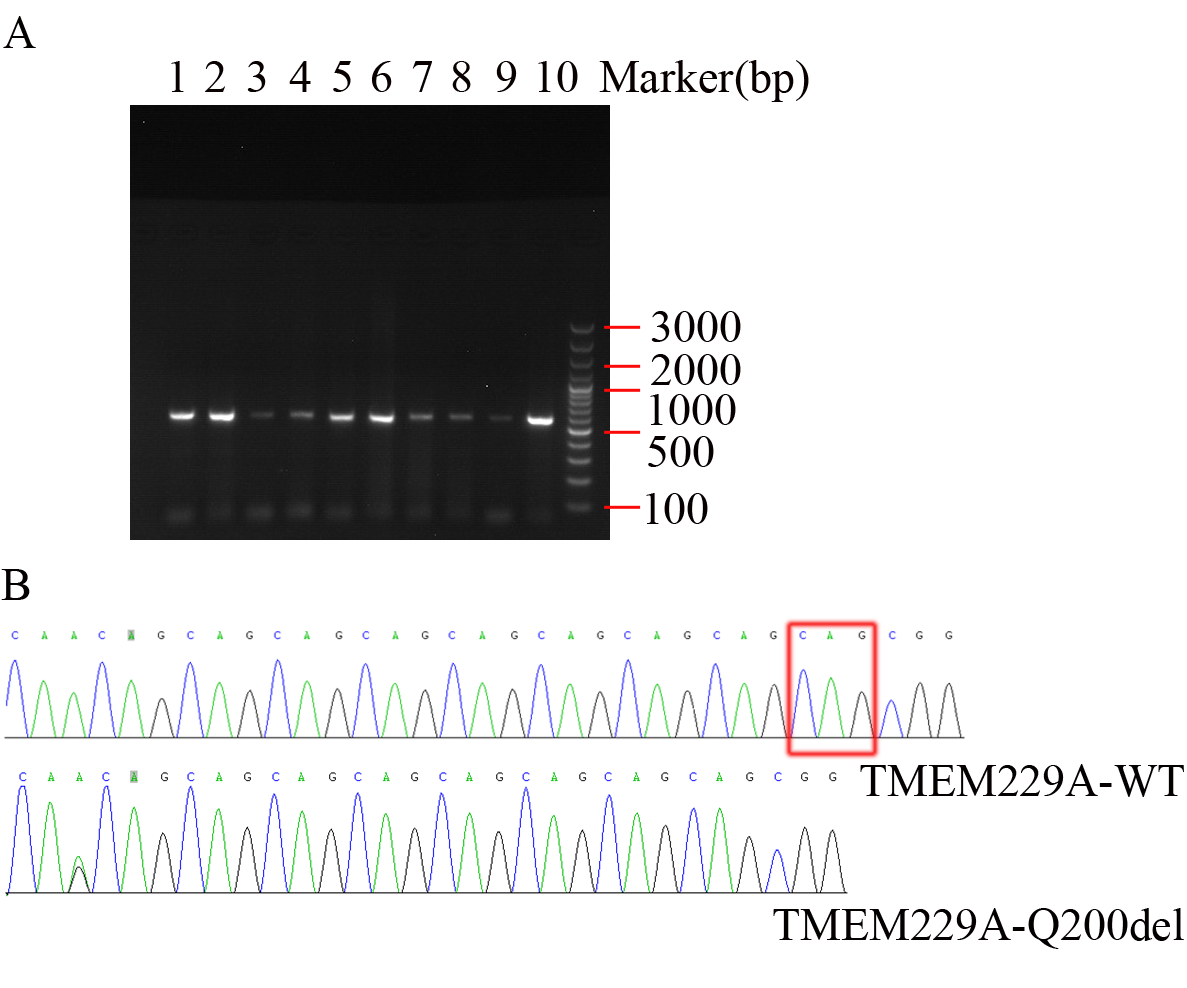

Supplement: Supplementary file 1 — Figure S1 TMEM229A Q200del genotype was identified. (A) TMEM229A Q200del mutation of selected 10 cases was identified using DNA electrophoresis; (B) TMEM229A genotype was further performed using DNA sequencing. [file CRJ-18-e70006-s001.tif]
